# Double Peripleuritis with Suppurative Mediastinitis

**Published:** 1882-10

**Authors:** 


### DOUBLE PERIPLEURITIS WITH SUPPURATIVE MEDIASTINITIS.

Peripleuritis is not described exactly in handbooks of pathology. It is an inflammation of the tissue which connects the pleura costalis with the ribs. It can be of a traumatic character, or it may have its origin in the pleura, or an alteration of the bone-substance of the ribs, and it may terminate in abscess. In all the published cases, as that of Dr. Muratti, the cause of the disease is similar to that of empyema. But there are two differential points in these two diseases. In empyema, the ribs and the intercostal spaces are uniformly extended in a large extent of space, while in peripleuritis but one or two intercostal spaces are enlarged. In empyema there is a purulent infiltration between all the pleural laminæ, while in peripleuritis there is but a localized cavity filled with pus. Percussion will show this exactly. The following case gives a true picture of the disease. The patient, thirty-six years of age, complained in the month of December, 1878, of a gnawing feeling and pain in the right side of the thorax, and on pressure of the postero-inferior part of this region the pain increased. Movement of the body did not hurt. In March, 1879, he noticed, beside the pain, a small blister with altered skin around it, which broke open and discharged some pus. Granulations sprang up, but the flow of pus continued. After a while the patient began to expectorate a sweet liquid, similar to that of the abscess. The fistulous opening was enlarged with the knife, and it healed quickly, but the pain reappeared. The patient entered the hospital in December, 1880. The thorax was cylindrical, with lateral deviations from the vertebral column; respiration bronchial. Under the seventh rib of the right side, four cm. from the vertebral column, there was a fistulous opening, the region around it being œdematous, and one point on the rib itself very painful. On the paravertebral region, in correspondence with the fistulous area, there was a very painful place. The vesicular murmur on the right side was very indistinct. The expectorated masses had a similar appearance and taste to that of the fistulous opening. There was further a sucking noise in the fistulous opening in expiration. The diagnosis was fistula of the bronchi caused by double peripleuritis and mediastinitis. An excision of the rib was made, but the patient died after a short time. The autopsy proved the correctness of the diagnosis.—*Gazetta Medica Italiana.*

